# The Gastroprotective Role of *Acanthus ilicifolius* – A Study to Unravel the Underlying Mechanism of Anti-Ulcer Activity

**DOI:** 10.3797/scipharm.1108-11

**Published:** 2012-06-18

**Authors:** K. T. Mani Senthil Kumar, Zothan Puia, Samir K. Samanta, Rajiv Barik, Arnab Dutta, Bapi Gorain, Dilip K. Roy, Dipan Adhikari, Sanmoy Karmakar, Tuhinadri Sen

**Affiliations:** 1 Department of Pharmaceutical Technology, Jadavpur University, Kolkata 700032, India.; 2 School of Natural Product Studies, Jadavpur University, Kolkata 700032, India.

**Keywords:** *Acanthus ilicifolius*, Sundarban mangroves, Ulcer protection, Dual inhibitor

## Abstract

*Acanthus ilicifolius* (Acanthaceae), a mangrove medicinal plant, is widely used by the local inhabitants of the Sundarbans (India) to treat a variety of diseases. As a part of our continued search for novel bioactive products from mangrove medicinal plants, we were able to document the anti-inflammatory effects of this plant. In the present study, we have performed a detailed evaluation of the gastroprotective activity of the methanolic extract of Acanthus *ilicifolius* using different models of gastric ulceration. Unlike the conventional non-steroidal anti-inflammatory drugs, a methanolic extract of *Acanthus ilicifolius* leaves (MEAL) possessing significant anti-inflammatory properties, as revealed from our previous studies displayed in rats in dosages of 200 mg and 400 mg/kg BW after intraperitoneal administration, showed significant protective activity (anti-ulcer activity) against the gastric lesions induced by aspirin, indomethacin, stress, ethanol, and pylorus ligation. In pylorus-ligated rats, administration of Methanolic extract of *Acanthus ilicifolius* leaves (MEAL) significantly decreased gastric volume, acidity, and peptic activity. Moreover, pre-treatment with MEAL significantly restored the levels of reduced glutathione (GSH) and the antioxidant enzyme superoxide dismutase (SOD), catalase (CAT), and glutathione peroxidase (GPX), along with significant inhibition of both lipid peroxidation and myeloperoxidase (MPO) activity in pylorus-ligated animals. Ulceration induced with ethanol was significantly inhibited with MEAL, and the extract also resulted in the reduction of both lipid peroxidation and myeloperoxidase activity. Furthermore, in this experimental model, administration of MEAL improved the activities of SOD, CAT, GSH, and GPX. A similar pattern of action was also noticed in cold-restraint stress-induced (CRS) ulceration, where MEAL pre-treatment inhibited CRS-induced ulceration, improved the status of antioxidant enzymes, and also reduced the level of lipid peroxides. These results suggest that extracts of the leaves of *Acanthus ilicifolius* may exhibit anti-ulcer activities additional to the anti-inflammatory properties.

## Introduction

*Acanthus ilicifolius* Linn. (Acanthaceae) is a spiny herb of the mangrove species, distributed widely throughout Southeast Asia. In India, the plant is found in the Sundarban mangroves and is used locally for the treatment of rheumatism, snakebite, paralysis, asthma, ulcers, and wound healing [1, 2]. The plant was reported for its analgesic, anti-inflammatory, anti-oxidant, hepatoprotective, and tumor-reducing properties [2–4]. The compound 2-benzoxazolinone isolated from the plant, exhibited significant anti-leishmanicidal activity [5]. The leaf extract was found to possess significant anti-cancer activity in transplantable tumor models and reduced the DNA alterations in murine liver [6]. The coumaric acid derivative, acancifoliuside, was isolated from the plant along with acteoside, isoacteoside, acanthaminoside, (+)-lyoniresinol 3α-*O*-β-glucopyranoside, (−)-lyoniresinol, and alpha-amyrin, of which the acteosoide, acanthaminoside, and (+)-lyoniresinol 3α-*O*-β-glucopyranoside were found to exhibit osteoblastic activity in cultured MC3T3-E1 cells [7]. Phytochemical studies with the plant revealed the presence of lignans, benzoxazinoid [8, 9], and megastigmane glycosides [10, 11].

A gastric ulcer is a common disorder, the pathophysiology of which has not yet been completely understood. This disease can arise frequently due to the irrational use of NSAIDs, stress, smoking, and alcohol consumption. Furthermore, oxidative damage is considered to be a common factor in the pathogenesis of ulceration observed from different experimental and clinical models. Different free radicals are known to be involved in the development of ethanol, indomethacin, paracetamol, and cold-restraint stress-mediated gastric mucosal damage [12–14]. Similarly, it has been observed that plants rich in polyphenols and various natural polyphenolics [15] demonstrate dual anti-inflammatory and anti-ulcer activities, through the inhibition/blockade of ROS generation [16].

Previous studies with an extract of the leaves of *Acanthus ilicifolius* indicated the presence of significant anti-inflammatory activity in different models of inflammation. The extract was also found to inhibit both cyclooxygenase (COX) and 5-lipoxygenase (5-LOX) activity, along with significant inhibition of cytokine generation [17]. In the present study, an attempt has been made to evaluate the effect of the methanolic extract of *Acanthus ilicifolius* leaves (MEAL) on different models of gastric ulceration.

## Experimental

### Plant Material

*Acanthus ilicifolius* Linn. leaves were collected from the Sundarban mangroves (West Bengal) in September 2006. The identification of the plant was performed by the Botanical Survey of India, Coimbatore, and a voucher specimen has been preserved for future use.

### Extraction

The leaves were thoroughly washed and dried in shade. The shade-dried leaves were reduced in size using a pulverizer, and passed through mesh to get uniformly coarse powder. The powdered material (3 kg) was extracted cold with petroleum ether to remove the fatty matter, followed by chloroform extraction. Then the cold extraction was carried out with methanol (99%) until complete exhaustion of the phytoconstituents. The extract was concentrated under reduced pressure (Rotary vacuum evaporator, Eyela SB-1100, Japan) to yield a greenish-black methanolic fraction (5.8 g), referred as MEAL, which was stored thereafter at 4°C until usage.

### Experimental animals

Male albino Wistar rats (175–200g) maintained under controlled uniform laboratory conditions were used for the study. The animals were fed with a standard Pellet diet (Lipton, India) and water ad libitum. The animals were housed under standard laboratory conditions. The animals had fasted for 24 h, prior to the study. The drugs were administered intraperitoneally (i. p.) unless otherwise mentioned. The experiments were performed in accordance with the care and use of experimental animals prescribed by the Institutional Animal Ethics Committee (IAEC), constituted under the guidelines of the Committee for the Purpose of Control and Supervision of Experimental Animals (CPCSEA), India.

### Analysis of the extract using HPLC

**Fig. 1:**
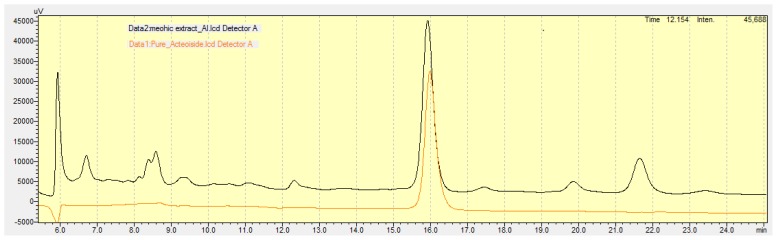
HPLC fingerprint of methanolic fraction of *Acanthus ilicifolius*, where the detection was carried out at 334 nm. The major peak (retention time ca. 16 min) corresponded to Acteoside.

The HPLC fingerprint (figure 1) was carried out using the Shimadzu LC 10 system with a pre-packed C 18 column [Phenomenex 5 μm particle size] having a security guard cartridge system and a UV detector. The chromatogram was carried out at 25°C under the following conditions: The mobile phase consisted of: solvent A (Water + 0.1% Trifluoro-acetic acid); solvent B (acetonitrile). Solvent A and B were mixed in the ratio of 80:20 and the flow rate was 0.6 ml/min). Acteoside was used as the standard. The peaks were detected at 334 nm.

### Acetyl salicylic acid-induced gastric lesion in rats

Male rats were divided into different groups comprising six animals each, which received either MEAL (200 and 400 mg/kg) or the vehicle or ranitidine 50 mg/kg. Half an hour later, acetyl salicylic acid (suspension in 1% carboxymethyl cellulose; 250 mg/kg) was administered orally to all the animals. Six hours later, the animals were sacrificed under ether anesthesia, the stomachs were removed, opened along the greater curvature, washed with normal saline, and the stomach tissues were examined under microscope for the ulcers produced. The length of the ulcer served as the ulcer index [18].

### Indomethacin-induced ulcer

Different groups of fasted rats (n=6) were treated with the control vehicle; standard drug – ranitidine 50mg/kg or MEAL 200 and 400 mg/kg, 30 minutes prior to indomethacin treatment (20 mg/kg; s.c.). The animals were sacrificed after a period of eighteen hours under ether anesthesia, and the stomachs were removed and washed with normal saline. Thereafter, the region of the stomach was microscopically examined for gastric ulcerations in the manner described previously [19]. The mucosal tissue was also used for the estimation of tissue lipid peroxidation (LPO) and the details are described below.

### Ethanol-induced ulcer

Groups of fasted male rats (n=6) were treated with the control vehicle; sucralfate (250 mg/kg p.o.) or MEAL (200 and 400 mg/kg), 30 minutes prior to administration of ethanol (50% ethanol; 5 mL/kg p.o.). The animals were sacrificed after one hour under ether anesthesia and the stomachs were washed with cold saline and examined for the ulcer index microscopically [20]. The stomach tissue was further used for the estimation of LPO, SOD, CAT, GSH, GPX, and MPO. The methodology for such biochemical studies are described below.

### Indomethacin–alcohol induced ulcer

Male rats fasted for 24 hours, were grouped (n=6), and were administered indomethacin (8 mg/kg s.c.) one hour prior to the control vehicle; ranitidine 50 mg/kg or MEAL (200 and 400 mg/kg); after 30 minutes ethanol (5 mL/kg, p.o.) was administered to all the groups. Seven hours later, under anesthesia, the animals were sacrificed and the stomachs were removed, washed with normal saline, and examined under microscope for the quantification of ulcer lesions [21]. The stomach tissue was also used for the quantification of tissue LPO as discussed below.

### Stress-Induced Gastric Ulcer

Stress-induced ulcers were induced according to the method of Senay and Levine [22]. Male rats fasted for 24 hours, were divided into different groups consisting of six rats per group. The animals treated either with the control vehicle (ranitidine 50 mg/kg) or MEAL (200 and 400 mg/kg) were restrained in their small cylindrical wire cages and placed in a cold chamber (2–4°C) for two hours. Then the animals were sacrificed under anesthesia and analyzed for the ulcer score as stated above. Further, the tissue samples were also utilized for the estimation of LPO as discussed below.

### Gastric secretory study in pylorus-ligated rats

Fasted rats were divided into different groups (n=6) and were administered with the control vehicle, MEAL 200 and 400 mg/kg or Ranitidine 50 mg/kg. After a period of 30 minutes, the animals were anaesthetised with ether, the abdomen was cut open, and the pyloric end was ligated without damaging the blood supply. The animals were sacrificed four hours after pylorus ligation, the stomach was removed and opened along the greater curvature and the ulcers generated were scored [23]. The gastric contents were collected, measured, and centrifuged at 2000 rpm for 10minutes. The supernatant was used for the determination of volume, pH, acidity, and peptic activity. The pH of the gastric juice was determined using standard pH paper (Merck. India). The acidity of the gastric juice (0.2 mL diluted to 2 mL) was determined by titration against 0.1 N NaOH using phenolphthalein as an indicator and expressed as mEq/L. The peptic activity was determined following the method of Debnath [24]. The gastric tissues were also used to form the estimation of hexosamine, mucus, LPO, GSH, SOD, catalase, myeloperoxide (MPO), and glutathione peroxidase. The details are discussed below.

### Estimation of biochemical parameters

The stomachs were washed with normal saline and cut into small pieces and homogenized in a Potter-Elvehjem glass homogenizer in ice cold 0.15 M KCl to obtain a 20% homogenate. This homogenate was used for the determination of different biochemical parameters such as hexosamine, LPO, GSH, SOD, CAT, GPX, and MPO.

### Assay of hexosamine

Hexosamine in the gastric mucosal tissue was assayed according to the method of Glick [25]. The gastric tissue homogenate (1 mL) was hydrolyzed in acid medium with 6 N HCl, and then the hydrolysate was neutralized with 3 N NaOH. One mL of the aliquot was mixed with 1 mL of acetyl acetone reagent (1 mL acetyl acetone in 50 mL of 0.5 N Na_2_CO_3_) and then mixed with Ehrlich’s reagent and the volume was made to 10 mL with 95% ethanol. The absorbance was measured at 530 nm.

### Assay of mucus content

The total mucus present in gastric tissue was estimated by measuring the amount of Alcian blue (AB) bound to mucus spectrophotometrically at 615 nm [26]. One milliliter of gastric tissue homogenate was incubated with AB reaction medium (1% AB and 0.16 M sucrose in 0.05 M sodium acetate, pH 5.8) for two hours. The mixture was centrifuged and the concentration of the Alcian blue in the supernatant was estimated spectrophoto-metrically, and was expressed in terms of Alcian blue bound per gram of glandular tissue homogenate.

### Determination of lipid peroxidation

The level of lipid peroxidation in the gastric tissue was determined by the method of Okhawa [27]. Briefly, the reaction mixture consisted of 0.2 mL of homogenate, 0.2 mL of 8.1 % SDS, 1.5 mL of 20 % acetic acid solution (adjusted to pH 3.5 with NaOH), and 1.5 mL of 0.8 % aqueous solution of TBA. The mixture was made for a final volume of 4 mL with distilled water, and heated in a water bath for 60 minutes. The reaction mixture was then added to a mixture of *n*-butanol and pyridine (15:1, v/v). The absorbance of the organic layer was measured at 532 nm. The malondialdehyde concentration was determined from the standard curve prepared with 1,1,3,3-tetraethoxypropane, and the values were expressed as nmol of TBARS/mg of protein.

### Determination of GSH

The analysis was performed following the method of Sedlak and Lindsay [28]. In brief, the specified volume of homogenized tissue samples were added to a solution containing 5% trichloroacetic acid and 5 mM EDTA at 4 °C, and centrifuged for 10 min at 15000g (4°C). The supernatant was added to 0.4 M Tris-EDTA buffer (pH 8.9) and then mixed thoroughly, and followed by the addition of 40 μL of 10 mM 3,3′-dithiobis(6-nitrobenzoic acid) (DTNB) in methanol. The absorbance was read at 412 nm after five minutes. The GSH concentration was determined from the standard curve of GSH and the values were expressed as μg/mg protein [29].

### Determination of superoxide dismutase (SOD) activity

SOD activity was measured as the inhibition of pyrogallol auto oxidation by the enzyme according to the method described by Marklund and Marklund [30]. The reaction mixture consisted of 0.05 mL of 0.2 mM pyrogallol solution in 10 mM HCl, tissue homogenate and 50 mM Tris-HCl buffer (containing 1 mM EDTA; pH 8.0) to make the final volume 2.5 mL. Auto oxidation of pyrogallol was measured by the increase in absorbance of the mixture at 420 nm (reading was taken at 30-second intervals for three minutes). A unit of enzyme is defined as the amount of enzyme that inhibits the reaction by 50%. Specific activity was expressed as unit/mg protein.

### Determination of catalase (CAT) activity

CAT activity was measured following the method of Beers and Sizer [31]. The tissue homogenate was mixed with H_2_O_2_ in a buffer solution (58.8 mM H_2_O_2_ in 0.05 M phosphate buffer pH 7) and the enzyme activity was measured at 240 nm (three minutes; 30-second intervals). Catalase activity was expressed as mM of H_2_O_2_ consumed/min/mg protein.

### Assay of glutathione peroxidase (GPX)

Glutathione peroxidase activity of the tissue homogenate was measured according to the method described by Lawrence and Burk [32]. The assay system contained the tissue homogenate along with 50 mM sodium phosphate buffer (pH 7.2), 1 mM EDTA, 1 mM sodium azide, 0.15 mM NADPH, 1 mM reduced glutathione, and 0.24 U of glutathione reductase. The reaction was initiated by adding 0.25 mM H_2_O_2_. The enzyme activity was monitored by the decrease in absorbance at 340 nm. The enzyme activity was expressed as μM of NADPH oxidized/min/mg protein.

### Assay of Myeloperoxidase (MPO)

Myeloperoxidase activity was measured following the method of Slungaard and Mahoney [33]. The assay system was prepared by mixing the homogenate with 50mM sodium acetate buffer (pH 5.25), 30 μM TNB, and150 mM NaCl. The reaction was initiated by the addition of 0.3 mM H_2_O_2_, and the decrease in absorbance at 412 nm due to oxidation of TNB (ɛ = 27,000 M^−1^ cm^−1^) was measured for one min. The activity of the peroxidase was expressed as nmoles of TNB oxidized/min/mg protein 13,000 M^−1^ cm^−1^.

### Statistical Analysis

Results were expressed as the mean ± S.E. Data were analyzed by one-way ANOVA followed by Dunnet’s post hoc test, and p<0.05 was considered to be statistically significant.

## Results

### HPLC analysis of MEAL

The HPLC fingerprint of the *Acanthus ilicifolius* methanolic fraction (MEAL) indicates the presence of five major peaks (measured at 334 nm). From the HPLC chromatogram, the major peak observed in the methanolic fraction was found to correspond to that of Acteoside (M.W. 624 a phenylpropanoid glycoside, purified from the same plant and used here as a chemical marker) at the retention time of 16 min (Fig. 1).

### Aspirin-induced ulcer

**Fig. 2:**
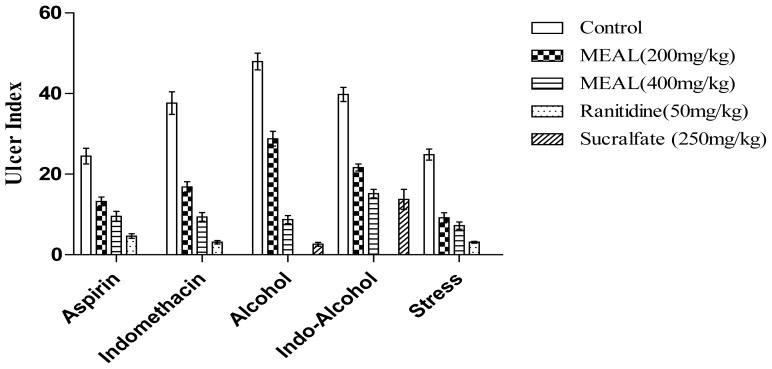
Effect of MEAL on gastric ulceration induced with different ulcerogens including Cold-restraint stress (CRS). Values are expressed as mean ± S.E.M; (n = 6); ** p < 0.01 (vs. Control)

MEAL at doses of 200 and 400 mg/kg significantly inhibited the ulceration in rats with respect to the control group. At the doses employed, MEAL inhibited the ulcer formation by 45.89% and 61%, respectively, whereas ranitidine at 50 mg/kg produced 81.12% inhibition. The gastric mucosa retained an almost normal appearance in the animals treated with MEAL, as compared to the control group as shown in Fig. 2.

### Indomethacin-induced ulcer

**Fig. 3:**
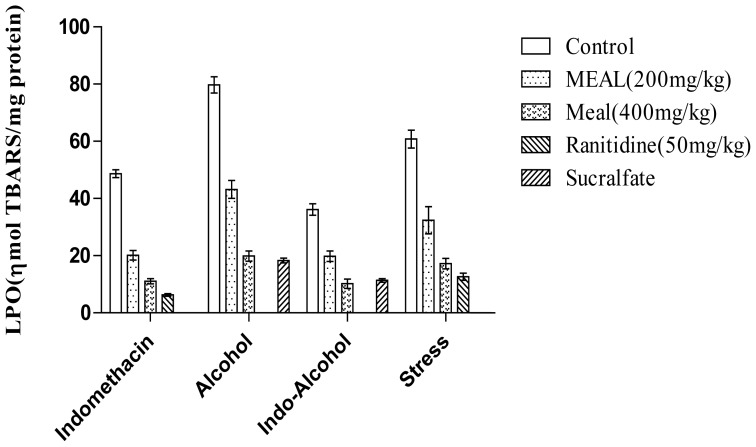
Effect of MEAL on tissue lipid peroxidation, estimated from the gastric tissues. Values are expressed as mean ± S.E.M; (n = 6); ** p < 0.01 (vs. Control).

Ulcer formation by indomethacin was significantly inhibited by pre-treatment with MEAL (200 and 400 mg/kg) and ranitidine 50 mg/kg. MEAL exhibited significant protection (55.23% and 75.03%) as shown in Fig. 2, whereas ranitidine showed better protective activity (91.73%). Moreover, it was also observed that MEAL (200 and 400 mg/kg) and ranitidine (50 mg/kg) attenuated lipid peroxidation by 58.65%, 77.26%, and 87.11%, respectively (Fig. 3).

### Ethanol induced ulcer

Pre-treatment with MEAL (200 and 400 mg/kg) and sucralfate (250 mg/kg) showed significant inhibitory activity against alcohol-induced gastric ulceration as shown in (Fig. 2). In ulcerated animals, the level of GSH, SOD, catalase, and GPX were found to be depleted when compared to the normal group of animals, whereas it was found to be restored in the animals treated with MEAL and sucralfate as shown in Table 1. In the control group of animals, the administration of alcohol produced significant activity in both LPO and MPO (Fig. 2). However, pre-treatment with either the test or the standard drug resulted in significant reduction of both tissue lipid peroxidation, as well as neutrophil myeloperoxidase activity (Table 1).

### Indomethacin–ethanol induced ulcer

Pre-treatment with indomethacin, followed by ethanol administration, produced severe hemorrhagic gastric lesions in the control group of animals. In animals treated with MEAL or the standard drugs, however, the ulcer formation was found to be significantly inhibited. The ulcer inhibition was found to be 45.61% and 61.87% in MEAL pre-treated animals (200 and 400 mg/kg) and 51.2% and 65.46% for animals treated with the drugs ranitidine (50 mg/kg) and sucralfate (250 mg/kg). Pre-treatment with either MEAL (200 and 400 mg/kg) or the standard drugs, produced significant inhibition of tissue lipid peroxidation (Fig. 2, 3).

### Stress-induced gastric ulcer

MEAL and ranitidine were found to significantly reduce the ulcer formation in the animals subjected to cold-restraint stress, when compared to the control group. A significant decrease in LPO levels was also found in animals pre-treated with either MEAL or ranitidine, when compared to the control group of animals (Fig. 2, 3).

### Gastric secretory study in pylorus-ligated rats

Pre-treatment with MEAL and ranitidine was found to significantly reduce the ulcer formation, volume, total acidity, and the peptic activity in the pylorus-ligated rats. Furthermore, it also produced a significant elevation of pH along with the elevation of mucus and hexosamine content (Table 2). Pre-treatment with the MEAL (200 and 400 mg/kg) and ranitidine (50 mg/kg) not only restored the status of various antioxidant enzymes (SOD, CAT, GSH, and GPX), but also suppressed tissue lipid peroxidation and caused a reduction in myeloperoxidase activity (Table 3).

## Discussion

Inflammation of the GI tract can affect the functioning of the mucosal barrier, thereby influencing its protective activity. Drug-induced damage to the GI tract has now become a global problem, due to widespread as well as indiscriminate use of NSAIDs. Therefore, effective management of GI ulceration would primarily depend on (i) reduction of the aggressive factors (ii) improved generation of protective factors or (iii) a combination of both. Advances in natural product chemistry have led to the purification and characterisation of a number of chemical compounds (alkaloids, saponins, triterpenes, tannins, etc.) with potent anti-ulcer activity [34]. In this study, it could be demonstrated that the methanolic extract of *Acanthus ilicifolius* leaves (MEAL) significantly reduced the formation of gastric ulcers in rats induced by various ulcerogens (aspirin, indomethacin, stress, ethanol, and pylorus ligation). A dose-dependent anti-ulcer activity, coupled with the restoration of altered biochemical parameters in the gastric tissue, was observed in the different models of ulceration.

Indomethacin, when administered prior to ethanol, led to the blockade of the COX pathway, thus shifting the arachidonic acid metabolism to the 5-LO pathway, which in turn led to enhanced production of leukotrienes (LTC_4_ and LTD_4_), leading to glandular disruption, excessive ulceration, and bleeding. Pre-treatment with 5-LO inhibitors effectively decreased such types of ulceration [35]. MEAL was found to act in a similar way. Administration of indomethacin or ethanol also led to enhanced ROS activity in the gastric mucosa, leading to enhanced lipid peroxidation. Pre-treatment of the animals with MEAL significantly reduced LPO in the gastric tissue, along with the restoration of GSH content.

Cold-restraint stress (CRS)-induced ulceration is known to occur due to the influence of both physiological and psychological factors [36]. According to reports, stress leads to increased gastric motility, vagal overactivity, mast cell degranulation, and decreased gastric mucosal blood flow [37]. According to some other studies, CRS can possibly lead to enhanced production of leukotriene-mediated histamine release [38], and also to decreased prostaglandin synthesis [39]. Interestingly in the current study, pre-treatment with MEAL produced significant inhibition of cold-restraint stress-induced ulceration in a manner similar to the naturally derived leukotriene antagonists and the 5-LO inhibitors silymarin [40], *Ocimum sanctum* [41], and *Boswellia serrata* [42]. Stress is also believed to cause mucosal ischemia, leading to increased generation of ROS and lipid peroxidation [43, 44]. Such increased activity of ROS often leads to mucosal damage with the subsequent destruction of epithelial basement membrane functioning [14, 45]. In our study, the level of lipid peroxides decreased following treatment with either MEAL or ranitidine, along with restoration of SOD and CAT levels. This decrease in the level of LPO may be attributed to the free radical-scavenging property of MEAL [17].

The gastric ulceration produced by pyloric ligation is caused by the increased accumulation of gastric acid and pepsin, leading to auto-digestion of gastric mucosa [46]. Defensive factors like the mucus and glycoproteins (hexosamine) are known to protect the submucosal layers from the back-diffusion of hydrogen ions [47, 48]. Administration of MEAL produced a significant increase in both mucus as well as hexosamine content in the tissue. MEAL significantly decreased the gastric volume, acidity, and peptic activity when compared to the control group of animals. Such a decrease in the gastric secretory activity could play an important role in the protection of the gastric mucosa.

Hence, dual inhibition of the COX-LOX [49] pathway could be advantageous in limiting inflammation when compared to the conventional NSAIDs; moreover, such a blockade of both pathways may also reduce incidences of gastric ulceration. In some instances, it has been observed that some drugs (natural or synthetic) not only produce dual inhibition of COX and 5-LOX, but also demonstrate free radical-scavenging properties and therefore, such substances can serve as a viable alternative to conventional NSAIDs. Drugs like Tepoxalin and Licofelone have been found to produce a dual blockade of both COX and 5-LOX enzymes, thereby displaying excellent anti-inflammatory properties coupled with an improved GI safety profile [50]. According to reports, Tepoxalin, a dual inhibitor, showed excellent gastric tolerance and it also counteracted the gastrointestinal side effects of indomethacin, when administered prior to indomethacin administration [51].

Earlier studies with *Acanthus ilicifolius* indicate the presence of different chemical substances [17], including acteoside (a major component found in the leaf extract), depicted in the HPLC analysis performed in our laboratory (Fig. 1). The leaf extract of this plant was found to produce significant COX and LOX (87 and 79%, respectively) inhibitory activity, along with potent free radical-scavenging properties [17]. Furthermore, pre-treatment with MEAL also improved the mucus content in the gastric tissue. Hence, it may be concluded that the effect of MEAL on gastric inflammation could probably be based on several mechanisms.

Thus it may be suggested that *Acanthus ilicifolius* leaf extracts possess anti-ulcer as well as anti-inflammatory properties. Drugs possessing such dual inhibitory effects may open up new perspectives in the effective management of acute and chronic gastric inflammatory conditions.

## Figures and Tables

**Tab. 1:** Effect of MEAL on antioxidant enzymes and myeloperoxide activity in gastric ulceration induced with alcohol

Treatment	Dose (mg/kg)	MPO (nmol/min/mg protein)	SOD (unit/mg protein)	CAT (mm of H_2_O_2_ consumed/min/mg protein]
Control	–	148.32 ± 12.36	20.57 ± 0.27	10.24 ± 0.31
MEAL	200	81.39 ± 7.32^**^	59.72 ± 0.84^**^	37.12 ± 0.21^**^
MEAL	400	60.76 ± 5.97^**^	77.13 ± 0.79^**^	49.32 ± 0.32^**^
Sucralfate	–	48.84 ± 1.73^**^	86.71 ± 0.93^**^	60.17 ± 0.76^**^

**Treatment**	**Dose (mg/kg)**	**GSH (μg/mg Protein)**	**GPX (μmole NADPH/min/mg protein)**	

Control	–	2.07 ± 0.36	4.36 ± 0.62	
MEAL	200	7.29 ± 0.53^**^	7.13 ± 0.52^**^	
MEAL	400	10.24 ± 0.36^**^	9.67 ± 1.23^**^	
Sucralfate	–	9.94 ± 0.87^**^	13.16 ± 0.73^**^	

**Tab. 2:** Effect of MEAL on ulcer index and gastric secretion following pyloric ligation in rats

Treatment	Dose (mg/kg)	pH	Ulcer Index Volume of Gastric (mm) Secretion (ml)
Control	–	1.21 ± 0.19	13.42 ± 1.48 9.86 ± 1.19
MEAL	200	2.43 ± 0.21**	6.59 ± 1.08** 5.14 ± 0.62**
MEAL	400	2.97 ± 0.36**	3.71 ± 0.72** 2.97 ± 0.36**
Ranitidine	50	3.51 ± 0.17**	1.13 ± 0.24** 1.84 ± 0.26**

**Treatment**	**Dose (mg/kg)**	**Total Acidity (mEq/L)**	**Peptic Activity (mg of tyrosine/ml)**

Control	–	68.76 ± 4.12	89.34 ± 4.13
MEAL	200	44.96 ± 3.86**	60.21 ± 3.62**
MEAL	400	29.11 ± 2.71**	47.21 ± 1.23**
Ranitidine	50	8.13 ± 1.82**	24.65 ± 2.97**

**Treatment**	**Dose (mg/kg)**	**Mucus Secretion (O.D. unit/mg Protein)**	**Hexosamine (μg/gm tissue)**

Control	–	0.13 ± 0.01	108.3 ± 2.87
MEAL	200	0.48 ± 0.023**	154.63 ± 4.28**
MEAL	400	0.56 ± 0.018**	179.87 ± 3.54**
Ranitidine	50	0.59 ± 0.014**	165.23 ± 4.74**

Values are expressed as mean ± S.E.M; (n = 6),

** p < 0.01 (vs. control).

**Tab. 3:** Effect of MEAL on various antioxidant enzymes, LPO and MPO in the gastric tissue of pylorus ligated rats

Treatment	Dose (mg/kg)	LPO (nmol of TBARS/mg protein)	MPO (nmol/min/mg protein)
Control	–	27.34 ± 1.23	164.2 ± 9.82
MEAL	200	14.32 ± 0.84**	90.73 ± 6.42**
MEAL	400	6.13 ± 0.67**	63.86 ± 7.21**
Ranitidine	50	4.13 ± 0.34**	46.21 ± 5.82**

**Treatment**	**Dose (mg/kg)**	**SOD (unit/mg protein)**	**CAT (mm of H_2_O_2_** **consumed/min/mg protein]**

Control	–	18.4 ± 0.42	4.56 ± 0.82
MEAL	200	74.9 ± 0.62**	10.18 ± 0.94^*^
MEAL	400	86.4 ± 0.49**	13.26 ± 1.11**
Ranitidine	50	94.3 ± 1.06**	15.27 ± 1.06**

**Treatment**	**Dose (mg/kg)**	**GSH (μg/mg protein)**	**GPX (μ mole NADPH/min/mg protein)**

Control	–	2.69 ± 0.14	3.79 ± 0.62
MEAL	200	8.45 ± 0.43**	9.41 ± 0.84^*^
MEAL	400	10.47 ± 0.36**	12.28 ± 1.41**
Ranitidine	50	12.04 ± 0.32**	14.59 ± 1.26**

Values are expressed as mean ± S.E.M; (n = 6),

** p < 0.01 (vs. Control).
